# Recombinant humanized collagen combined with nicotinamide increases the expression level of rat basement membrane proteins and promotes hair growth

**DOI:** 10.3389/fbioe.2025.1546779

**Published:** 2025-06-12

**Authors:** Youshiqi Zhou, Boyu Chen, Shijia Ye, Haoyu Sun, Shuyue Wang, Xiaozhen Diao, Wenhui Wu

**Affiliations:** ^1^ Department of Marine Biopharmacology, College of Food Science and Technology, Shanghai Ocean University, Shanghai, China; ^2^ Putuo Sub-Center of International Joint Research Center for Marine Biological Sciences, Zhoushan, China; ^3^ Marine Biomedical Science and Technology Innovation Platform of Lin-gang Special Area, Shanghai, China

**Keywords:** recombinant humanized collagen, hair follicle stem cells, hair loss, VEGF, trichohyalin

## Abstract

**Background:**

Hair follicle stem cells (HFSCs) play crucial roles in hair growth and are expected to be potential targets in regenerative medicine and tissue engineering.

**Method:**

This study aims to investigate the positive effect on hair growth by the recombinant human collagen complex (RHC complex), composed of rhCOL III, rhCOL XVII, and rhCOL XXI, along with nicotinamide, both *in vitro* and *in vivo*, by HFSCs and rat models. The survival rate, function, and differentiation of HFSCs were investigated.

**Results:**

The CCK-8 experiment showed that the RHC complex was non-toxic to HFSCs, and the cell survival rate exceeded 80% after 8 and 16 h of treatment. The ELISA method showed that the RHC complex significantly increased the intracellular vascular endothelial growth factor (VEGF) levels. In addition, the increase in the content of trichohyalin (a key structural protein of hair) indicates that the structure and function of hair follicles may be enhanced. The expression levels of β-integrin and p63 were significantly upregulated, which are crucial for cell adhesion, migration, and maintenance of HFSCs homeostasis. In the rat model, significant hair growth was observed after a 7-day treatment period. The period of vigorous hair growth in rats was selected for immunofluorescence, enzyme-linked immunosorbent assay (ELISA) and hematoxylin-eosin (HE) staining analysis. The results showed that the RHC complex could promote the expression of Integrin, Laminin and Perlecan, which were conducive to maintaining the stability of the microenvironment of HFSCs. Additionally it facilitated the migration and differentiation of HFSCs, as evidenced by an increased number of hair follicles in HE-stained skin tissues. In conclusion, the RHC complex exhibited high HFSCs survival rates and enhanced the expression of HFSCs-associated factors and basement membrane proteins. These properties make the RHC complex a promising novel ingredient for promoting hair growth, preventing hair loss, and maintaining hair health.

## Graphical Abstract

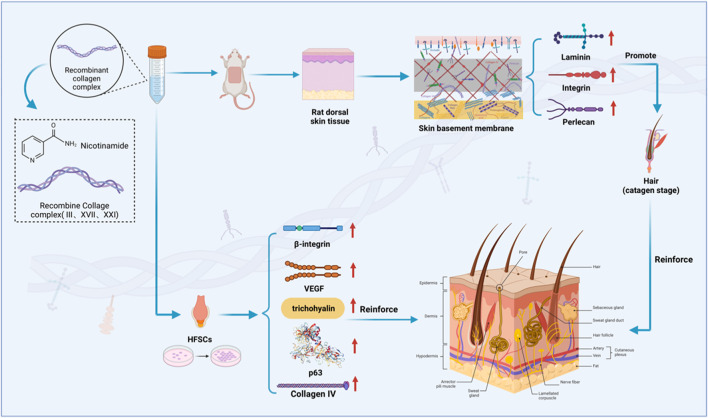

## 1 Introduction

Androgenic alopecia (AGA) is a common skin disease characterized by progressive hair loss, which is increasingly affecting young people worldwide (Guo and Katta, 2017). The current treatment methods, including minoxidil, finasteride, and hair transplantation, face limitations such as short-term efficacy, systemic side effects, or invasive surgery (Sonthalia, 2016). Therefore, it highlights the urgent need for safer and more effective treatment strategies (Sica, 2004).

Hair follicle stem cells (HFSCs) are adult stem cells located in the protruding part of hair follicles and have become a promising new target for hair growth (Sun et al., 2024; Zhang and Chen, 2024). In 2020, Kim et al. demonstrated that stimulating HFSCs can initiate the growth cycle of hair follicles, thereby alleviating hair loss (Kim et al., 2020). The emerging evidence emphasizes the crucial role of the HFSCs niche, particularly the basement membrane (BM) microenvironment, in regulating hair follicle circulation and stem cell homeostasis (Xing et al., 2024; Liu et al., 2022a). BM is a special extracellular matrix (ECM) structure rich in type IV collagen, laminin, and nestogens, providing necessary structural support and biochemical clues for hair follicle morphogenesis and hair growth (Raja et al., 2023; Stanley et al., 1982). Hair keratin related proteins, such as trichohyalin, strengthen the mechanical strength of the inner root sheath through cross-linking networks (Steinert et al., 2003), integrins and TGF - β mediate mechanical transduction and epithelial mesenchymal crosstalk (Watt and Fujiwara, 2011), which is crucial for hair growth and structure.

Collagen is the structural backbone of the hair follicle ECM. Due to reduced synthesis and degradation mediated by MMPs, collagen gradually decreases with age, thereby damaging the integrity of the hair follicle (Brennan et al., 2003). Postlethwaite et al. observed that collagen peptides are transported through the bloodstream to the skin and accumulated there, indicating a potential mechanism for supplementing collagen in the skin and maintaining its structural integrity (Yazaki et al., 2017). Although collagen peptides have shown potential in supplementing skin collagen, animal derived collagen has immunogenicity and the risk of pathogen transmission (Bai et al., 2023; You et al., 2023). Recombinant humanized collagen protein (RHC) engineered through microbial expression systems provides a pathogen free alternative with adjustable biological activity (Liu et al., 2024). It is worth noting that type XXI collagen (COL21A1) is a fibroblast associated collagen with interrupted triple helix (FACIT), which has recently received attention for its regulatory role in ECM remodelling and tissue repair (Kehlet and Karsdal, 2016).

Currently, relevant studies have shown that the synergistic effect of type III and type XVII collagen can effectively promote hair growth (Liu et al., 2022b; Jufang, 2024). In 2002, Elise A. Olsen et al. found that niacinamide, even as a placebo, still has a positive effect on hair growth (Olsen et al., 2002). Therefore, based on this result, we propose a hypothesis that the collagen complex composed of nicotinamide and rhCOL III, rhCOL XVII, and rhCOL XXI may synergistically enhance BM protein synthesis, thereby stabilizing the hair follicle niche and having beneficial effects on hair growth and health. This study utilized *in vitro* cell models and rat skin models to evaluate the potential of the RHC complex in promoting hair growth.

## 2 Methods

### 2.1 Chemicals and reagents

Trypsin-EDTA was purchased from Gibco (Grand Island, NY, United States). Phosphate buffer solution (PBS) was obtained from Servicebio Technology (Wuhan, Hubei, China). CCK-8 was obtained from AbMole BioScience (Harvard, TX, United States). Collagen IV antibody from rabbit was purchased from Affinity (Bioscience, Harvard, CA, United States) and DyLight 594, goat anti-rabbit IgG was purchased from Abbkine (Wuhan, China).

### 2.2 HFSCs preparation and cell culture

Mouse Hair Follicle Stem Cells (HFSCs) and a complete medium were purchased from Pricella Biotechnology (Nanjing, China) Mouse hair follicle stem cells were cultivated in a complete culture medium containing 10% FBS and 1% P/S solution at 37°C with 5% CO_2_ cultivate and passage the primary cells and then use the seventh-generation cells for the following experiments. HFSCs were reseeded in a 96-well plate (Corning, NY, United States) at a density of 1 × 10^4^ cells/well for the CCK-8 assay and in a 12-well plate (Corning, NY, United States) at a density of 1 × 10^5^ cells/well for the enzyme-linked immunosorbent assay (ELISA).

### 2.3 Recombinant humanized collagen complexes synthesis

Recombinant humanized type III collagen (rhCOL III, Mw: 10–43 kDa), recombinant humanized type XVII collagen (rhCOL XVII, Mw: 10–23.8 kDa), recombinant humanized type XXI collagen (rhCOL XXI, Mw: 10–38 kDa) were provided by Chengdu Zhanyan Biotechnology Co., Ltd. And the amino acid sequence of three proteins was shown in the Supplementary Table S1. Nicotinamide was provided by Shanghai Macklin Biochemical Technology Co., Ltd. Recombinant collagen was dissolved in deionized water at a ratio of rhCOL III: rhCOL XVII: rhCOL XXI: Nicotinamide = 400:100:50:2.

### 2.4 CCK-8 assay

HFSCs suspensions (100 μL, 1 × 10^4^ cells/well) were added to a 96-well plate (Corning, NY, United States) at 37°C in a 5% CO_2_ incubator. After corresponding treatments for different groups, cells were treated with 10 µL of CCK-8 solution (AbMole, Bioscience, Harvard, TX, United States) at 37 °C for 1h. The optical density was measured at a wavelength of 450 nm using a microplate reader (Agilent, Santa Clara, CA, United States).

### 2.5 Rat hair removal model preparation

Wistar rats were purchased from Shanghai Slac Laboratory Animal Co. Ltd., Shanghai, China. Animal study protocols and procedures were approved by the Shanghai Ocean University institutional animal care and use committee (Permit Number: SHOU-DW-2022-012). All methods were employed in accordance with the relevant guidelines and regulations of Scientific and Ethical Care and Use of Laboratory Animals of Shanghai Ocean University.

The hair on the back of each rat was shaved off using a skin preparation knife over an area of approximately 2 × 2 cm (4 cm^2^), followed by the application of depilatory cream to remove any remaining hair. After a recovery period of 24 h, thirty-five rats were randomly assigned to one of seven groups: 2 mg/mL, 0.5 mg/mL, 0.1 mg/mL, 0.05 mg/mL, 0.01 mg/mL, 0 mg/mL and control 5%Minoxidil.

### 2.6 Methods of administration

The corresponding concentration of rhCOL III: rhCOL XVII: rhCOL XXI: Nicotinamide = 400: 100: 50: 2. 120 μL was uniformly applied to the dorsal depilated area of rats in each group every day, and the day of administration was defined as the first day of the test, and it was applied once a day for 14 days. On days 7 and 14, respectively, the rats were euthanized, and the dorsal skin tissues were excised for histological analysis (Kim et al., 2021).

### 2.7 Documentation of hair growth status in rats

The hair growth status of the rats was observed and recorded every day, and the skin on the back of the rats was photographed and recorded on days 7 and 14 to compare the hair growth of different groups of animals during the 14 days.

### 2.8 Histologic observation of hair follicles (H&E)

At the end of the experiment, rat dorsal skin models were fixed in 10% formalin at 4°C overnight and dehydrated in a graded ethanol series for paraffin embedding. Section 3 mm thick were cut and mounted on slides. Tissue sections were deparaffinized in xylene and rehydrated in a decreasing graded ethanol series and then stained with hematoxylin and eosin (H&E). Stained slides were photographed through a microscope (Olympus, Tokyo, Japan) that was visualized and observed using Slide Viewer (3DHISTECH Ltd., Budapest, Hungary; version 2.6.0) software. Subsequently, for each sample, the three regions with the highest number of hair follicles were selected photographed and counted at ×100 magnification.

### 2.9 Immunofluorescent staining microscopy (IF)

After the monolayers were pretreated with collagen complex solution (0.01, 0.05, 0.1, 0.5, 2 mg/mL) or without (blank, NC) for 8–16 h. The monolayers were fixed in paraformaldehyde at 4°C for 30 min and rinsed three times with PBS buffer solution (PBS, Servicebio, Wuhan, China) for the immunofluorescent staining microscopy. The monolayers were then blocked with goat serum-blocking buffer for 30 min at room temperature. Collagen IV antibodies were used overnight at 4°C, followed by the 2nd antibody (DyLight594, goat anti-rabbit IgG antibody) in the dark at room temperature. A fluorescence microscope (BX53F2, Olympus, Tokyo, Japan) was used to observe the expression of the protein. Using ImageJ (version 1.46, NIH), three immunostained photomicrographs were randomly selected from each group and semi-quantitatively measured based on the proportion of target protein-positive areas.

Paraffin tissues were fixed with 4% paraformaldehyde (PFA) for 30 min, blocked with 3% bovine serum albumin (BSA) and PBS solution, incubated overnight at 4°C with Laminin beta 1 antibody (23498-1-AP, proteintech), Integrin alpha five antibody (ab150361, Abcam), Heparan Sulfate Proteoglycan 2 antibody (ab315029, Abcam) was incubated at 4°C overnight. After washing, the cells were incubated with Anti-rabbit lgG (H + L) secondary antibodies (#4412, CST) for 50 min and the nuclei were stained with DAPI (Beijing Boosun Biotechnology Co., Ltd., China). Laminin, integrin and perlecan protein expression was observed by immunofluorescence microscopy (OLYMPUS, Japan).

### 2.10 Enzyme-linked immunosorbent assay (ELISA)

Inoculate HFSCs (1 × 10^5^ cells/well) into a 12-well plate (Corning, NY, United States) and culture for 24 h. Discard the old culture medium and rinse with PBS. Except for the blank group, the other groups were treated with collagen complexes for 8–16 h. Wash off residual culture medium with precooled PBS. Add cell lysis buffer, let it sit on ice for 15 min, scrape off the cells, and centrifuge at 12,000 rpm at 4°C for 10 min. Take the supernatant and store it at −80°C. According to the instructions of the ELISA kit (Jiangsu, China), detect the levels of VEGF, p63, trichohyalin, and β - integrin. Add the diluted supernatant and standard solution of the upper layer cells to the pre-coated enzyme plate and react at 37°C for half an hour. Wash the plate five times, add enzyme reagent, and react at 37°C for half an hour. Wash the board five times and add colorimetric solution A and colorimetric solution B. Incubate at 37°C for half an hour and quickly add the termination solution. Measure the optical density at 450 nm using an ELISA plate reader. All ELISA results are expressed as the mean plus or minus the standard deviation (SD) of repeated measurements.

Skin tissue samples of rats in each group were taken, cut and weighed 0.2 g, then added with 1.8 mL PBS, and fully homogenized on the ice bath using a hand-held homogenizer. And the cultured medium was clarified by centrifugation at 10,000 rpm for 15 min at 4°C. The supernatant is obtained and placed on the ice to be measured. The supernatant was used to assess the level of laminin, integrin and perlecan using an ELISA assay kit (Jiangsu, China), following the manufacturer’s instructions.

### 2.11 Hair scoring system

Physically quantify the luster, growth rate, roughness, and density of rat hair treated with different concentrations of RHC complex. Refer to the ISO 17751 series standards and ASTM D3991-06 standards, and develop scoring criteria based on the physiological characteristics of animal hair. The total score is 12 points, with a maximum score of 3 points for hair luster, growth rate, roughness, and density. The evaluation table is shown in Table 1. Three parallel experiments are conducted each time, and the average value is taken as the scoring standard after data statistics.

**TABLE 1:** The quantify physical of hair growth.

Evaluation project	Evaluation criteria	Score value	Full marks
Hair luster	Matte: weak reflection, dull, dry, matte, rough	0	3
	Weak luster: specific angles reflection, uneven distribution, local dullness/yellowing	1	
	Medium luster: obvious reflection, even coverage, soft coherent, no dark areas	2	
	Strong luster: strong uniform reflection, mirror-smooth, all angles bright, high transparency	3	
Hair growth rate	Stagnation:≤ 0.5 cm/month	0	3
	Slow:0.5–1.0 cm/month	1	
	Normal:1.0–1.5 cm/month	2	
	Fast:≥ 1.5 cm/month	3	
Roughness of hair	Extremely rough: with obvious frizz, many raised hair scales, and split hair	0	3
	Rough: The hair scales are partially raised, the tips of the hair are slightly forked, and the luster is low	1	
	Smooth: The hair scales are basically closed, the surface is flat, and the luster is moderate	2	
	Extremely smooth: The scales are completely closed, the mirror surface is smooth, and the luster is strong and uniform	3	
Hair density	The epidermis is clearly visible, with sparse hair and clear exposed hair follicles in certain areas	0	3
	The epidermis is faintly visible, with wider hair gaps and overall hair volume less than other rats	1	
	The epidermis is not visible, the hair is evenly distributed, and the hair volume is moderate	2	
	The hair is thick and dense, completely covering the epidermis, and the diameter of the hair bundle is thick and robust	3	

### 2.12 Statistical analysis

Data were represented as mean ± standard deviation (SD). Statistical analysis was performed using one-way ANOVA or T-tests. Multiple comparisons of means were performed compared with the 1 μΜ minoxidil group using Fisher’s least significant difference (LSD) and Duncan. Statistical significance was set at *p < 0.05, **p < 0.01, ***p < 0.001 compared with the blank group; *#*p < 0.05, *##*p < 0.01, *###*p < 0.001 compared with 1 μΜ minoxidil group. All statistical analyses were performed using SPSS 27.0. Mapping was conducted using GraphPad Prism (La Jolla, CA, United States; Version 10) (Kim et al., 2021).

## 3 Results

### 3.1 The incorporation of nicotinamide

**FIGURE 1:**
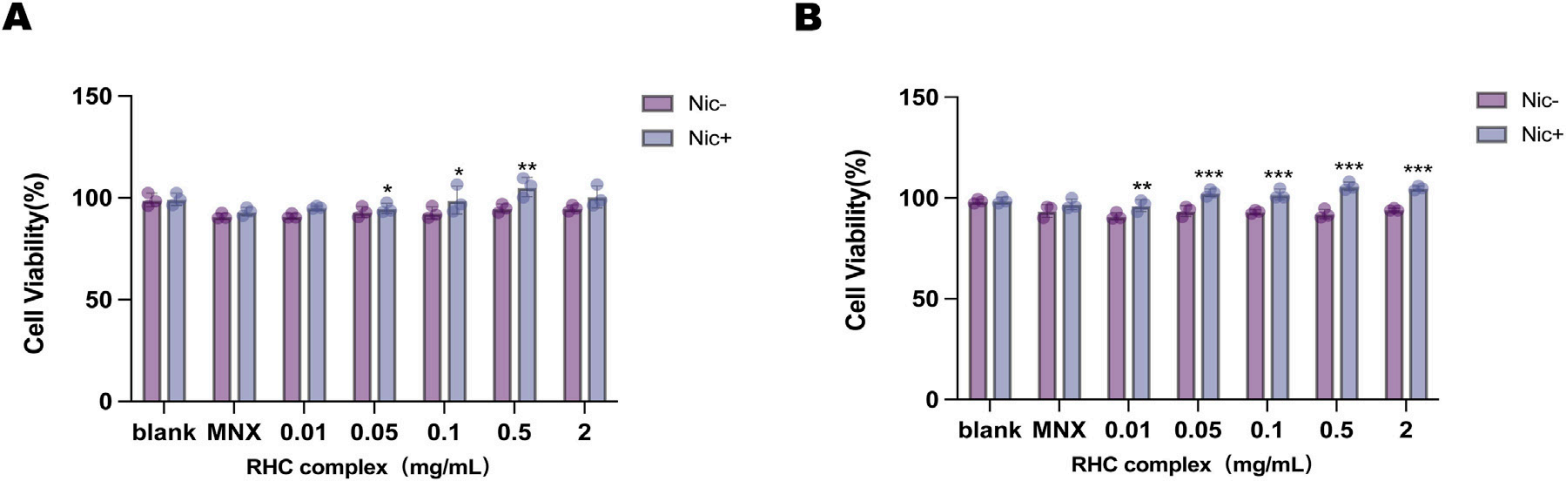
Different concentrations of RHC complex were cultured with HFSCs for 8–16 h **(A)** HFSCs viability was assessed via CCK-8 assay (Cell Counting Kit-8) following 8-h treatment with/without nicotinamide and the RHC complex. **(B)** HFSCs viability was assessed via CCK-8 assay (Cell Counting Kit-8) following 16-h treatment with/without nicotinamide and the RHC complex. The results are expressed as mean ± SD of three independent experiments. (*p < 0.05, **p < 0.01, ***p < 0.001 compared with the blank group).

HFSCs were cultured using the RHC complex with nicotinamide and without nicotinamide to observe cell viability. Figure 1A shows that after normalization with the blank group (0 mg/mL), the cell viability rate of complete medium containing different concentrations of RHC complexes (0.01, 0.05, 0.1, 0.5 and 2 mg/mL) after 8 h of treatment of both Nic - and Nic + groups remained greater than 80%. When the concentration of RHC complex was 0.5 mg/mL, the cell viability of Nic + group was significantly higher than that of Nic-group. When the concentration was 2 mg/mL, the cell viability of Nic + group was also significantly higher than that of Nic-group. Interestingly, for the 16 h administration (Figure 1B), at all concentrations, the cell viability of both Nic- and Nic + groups remained high, suggesting that the RHC complex still exhibited relatively low cytotoxicity after prolonged exposure.

### 3.2 The RHC complex improve the activity of HFSCs

**FIGURE 2:**
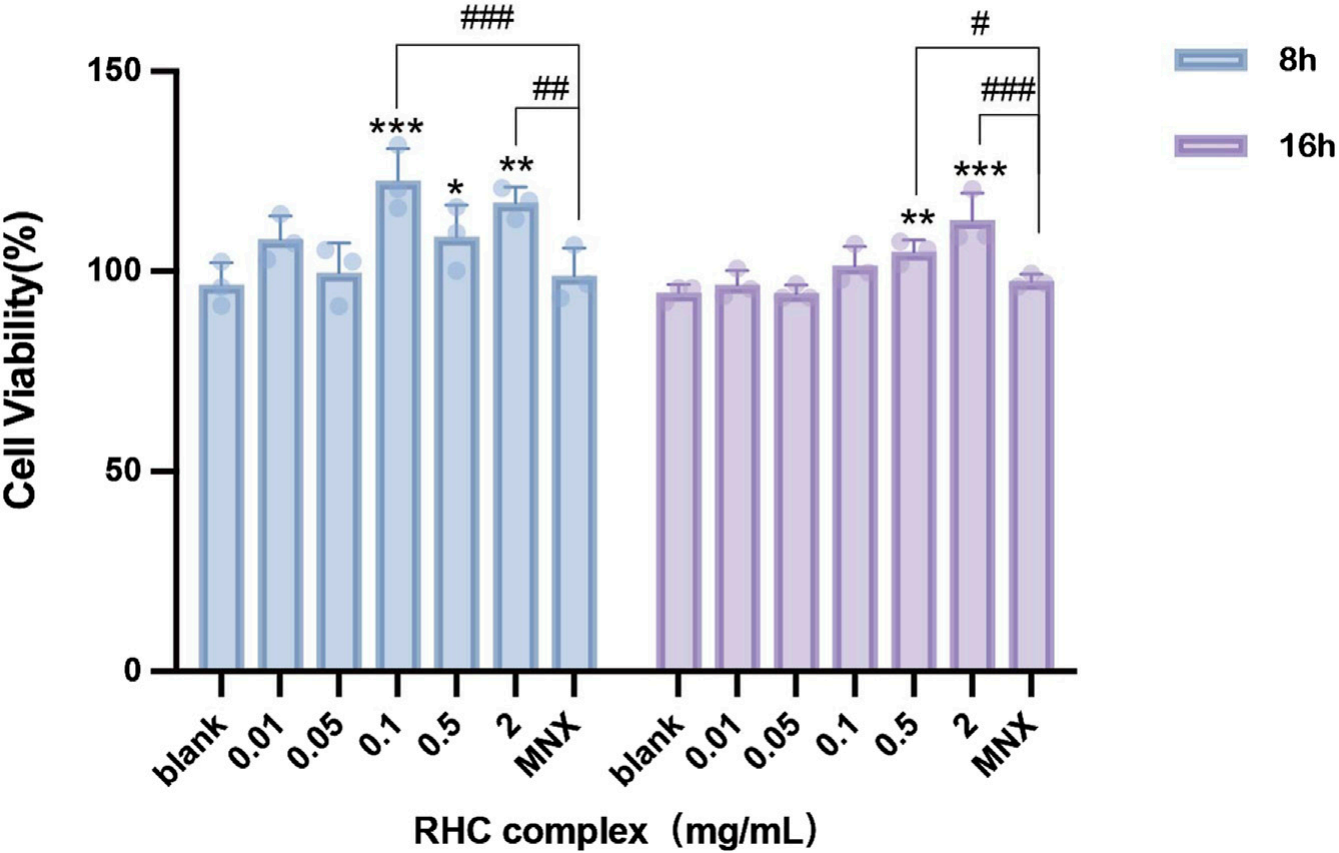
Different concentrations of RHC complex were cultured with HFSCs for 8–16 h. The cell viability of HFSCs was measured after treatment with RHC complex using a cck-8 assay. The results are expressed as mean ± SD of three independent experiments. (*p < 0.05, **p < 0.01, ***p < 0.001 compared with the blank group; #p < 0.05, ##p < 0.01, ###p < 0.001 compared with 1 μΜ minoxidil group).

HFSCs play significant roles in promoting hair growth. We treated these cells with various concentrations of RHC complex for 8–16 h. As shown in Figure 2, the cell viability rate of the combination of rhCOL III, rhCOL XVII, rhCOL XXI and nicotinamide was greater than 80%. Compared with the blank control group (0 mg/mL), there was a certain fluctuation in cell survival rate when the administration time was 8 h, but it was still higher or comparable to the control group, and the highest cell survival rate was observed at a concentration of 0.1 mg/mL of the RHC complex. After 16 h of administration, the survival rate of the drug concentration group significantly increased and showed concentration dependence. This was demonstrated by the fact that the survival rate of the RHC complex group gradually increased with increasing concentration of the administered drug and was higher than or comparable to that of the blank group (0 mg/mL) and the control group, and reached the highest value when the concentration of the RHC complex reached 2 mg/mL.

### 3.3 The RHC complex enhanced the expression of VEGF, P63, trichohyalin and β-integrin

**FIGURE 3:**
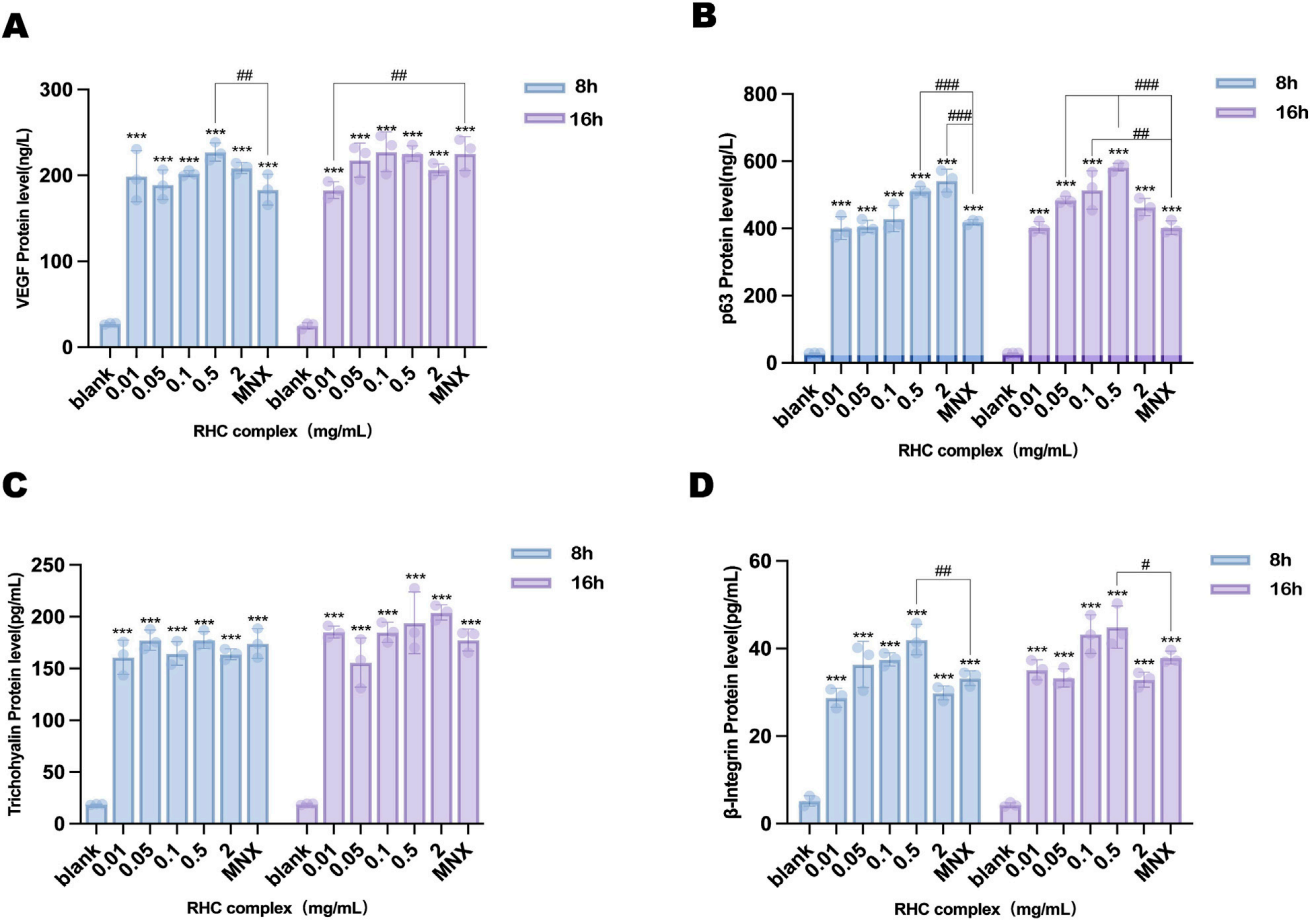
The levels of different concentrations in HFSCs treated with RHC complex (0.01, 0.05, 0.1, 0.5, 2 mg/mL) for 8–16 h were measured using ELISA assay. **(A)** determination of VEGF level in HFSCs. **(B)** determination of p63 level in HFSCs. **(C)** determination of trichohyalin level in HFSCs. **(D)** determination of β-integrin level in HFSCs. The results are expressed as mean ± SD of three independent experiments. (*p < 0.05, **p < 0.01, ***p < 0.001 compared with the blank group; ##p < 0.01, ###p < 0.001 compared with 1 μΜ minoxidil group).

The RHC complex significantly upregulated VEGF protein levels in HFSCs as determined by ELISA (Figure 3A). At concentrations of 0.05 and 0.1 mg/mL, VEGF levels at 16 h markedly exceeded those at 8 h, and the highest VEGF content (227.46 ± 23.18 ng/L) was achieved after 16 h of treatment with 0.1 mg/mL RHC complex. However, at a concentration of 2 mg/mL, no significant difference was observed between the levels of VEGF protein at 8 h and 16 h.

Similarly, p63 protein expression in HFSCs was elevated by the RHC complex (Figure 3B). While no time-dependent difference was observed at 0.01 mg/mL, concentrations of 0.05, 0.1, and 0.5 mg/mL resulted in significantly higher p63 levels at 16 h compared to 8 h. The peak intracellular p63 expression (582.32 ± 8.93 ng/L) occurred in the 0.5 mg/mL group after 16 h.

Trichohyalin levels exhibited distinct temporal patterns (Figure 3C). At 0.05 mg/mL, trichohyalin was significantly higher at 8 h than at 16 h. Conversely, concentrations of 0.01 mg/mL and 0.1–2 mg/mL showed elevated trichohyalin levels at 16 h, with the maximum concentration (204.14 ± 6.61 pg/mL) observed after 16 h of treatment with 2 mg/mL RHC complex.

β-Integrin expression demonstrated progressive enhancement over time (Figure 3D). All treatment groups exhibited significantly higher β-integrin levels compared to the control. Specifically, the 0.5 mg/mL group showed intracellular β-integrin contents of 42.04 ± 3.29 pg/mL at 8 h and 44.91 ± 4.38 pg/mL at 16 h, indicating sustained upregulation with prolonged exposure.

### 3.4 Enhancement of collagen IV expression in HFSCs

**FIGURE 4:**
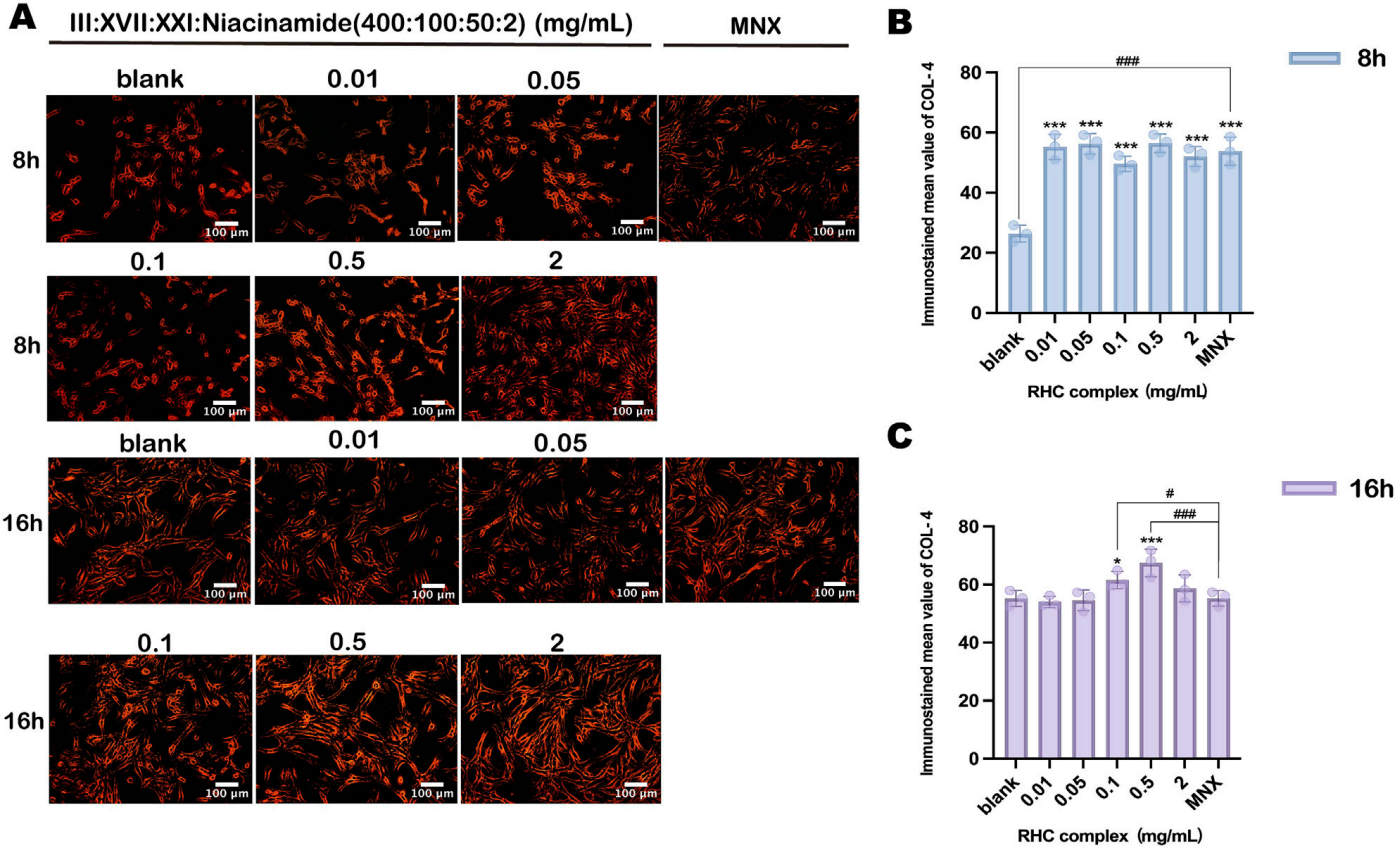
The expression of collagen IV in HFSCs induced by different concentrations of RHC complex. **(A)** Fluorescence microscopy observation images. Each image represents three similar experiments. (Scale bar: 100 µm). **(B)** Quantitative analysis of collagen IV immunofluorescence images after 8h of administration. **(C)** Quantitative analysis of collagen IVimmunofluorescence images after 16 h of administration. The results are expressed as mean ± SD of three independent experiments. (*p < 0.05, **p < 0.01, ***p < 0.001 compared with the blank group; #p < 0.05, ##p < 0.01, ###p < 0.001 compared with 1 μΜ minoxidil group).

Collagen IV(COL IV) was selected as the target protein to evaluate the efficacy of the RHC complex (rhCOL III: rhCOL XVII: rhCOL XXI: Nicotinamide = 400:100:50:2). Immunofluorescence analysis revealed concentration and time-dependent differences in COL IV expression among treated groups (Figure 4A). Quantitative analysis of immunostaining intensity demonstrated that all RHC concentrations exhibited comparable COL IV expression to the blank control (0 mg/mL) after 8 h, with concentrations of 0.01 mg/mL and 0.05 mg/mL showing significantly higher levels than the control (Figure 4B). Notably, prolonged exposure to the RHC complex for 16 h resulted in increased and more stable COL IV expression (Figure 4C), with expression levels surpassing those observed at 8 h, demonstrating a time-dependent amplification of synthesis. Importantly, the RHC complex exhibited comparable efficacy to minoxidil (positive control) in upregulating COL IV. These findings suggest that the RHC complex enhances COL IV expression in HFSCs, potentially stabilizing the extracellular matrix microenvironment to support hair follicle growth, with quantitative analysis further confirming its dose and duration-dependent effects.

### 3.5 The RHC complex promoted hair growth

**FIGURE 5:**
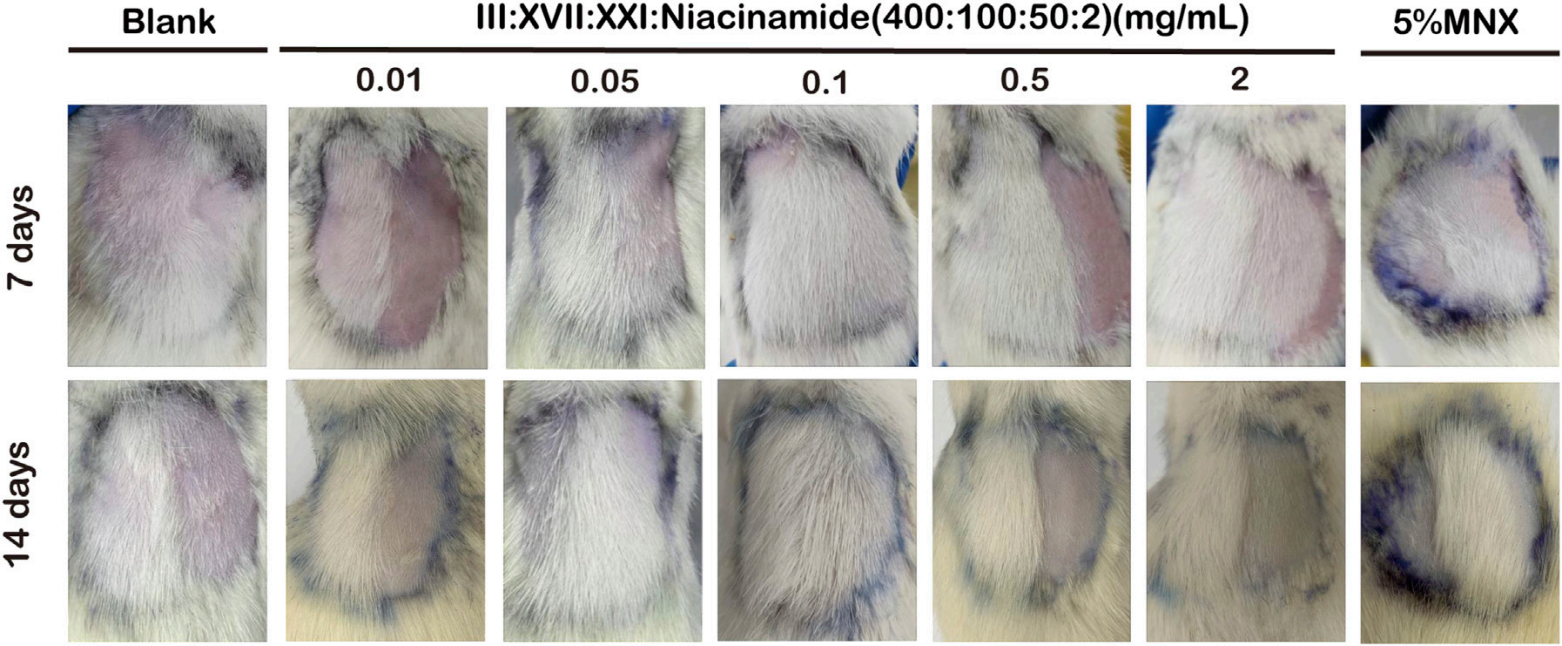
The dorsal skin of rats was treated with different concentrations of the RHC complex (rhCOL III: rhCOL XVII: rhCOL XXI: niacinamide = 400:100:50:2), which was administered at 24h intervals. Changes in hair growth were observed daily and photographs were taken on days 7 and 14.

In order to observe the effects of different concentrations of RHC complex on hair growth, a rat back hair loss model was established, and each group of rats was coated with 120 μL of RHC complex (at concentrations of 0.01, 0.05, 0.1, 0.5, and 2 mg/mL) every 24 h, and the drug was administered continuously for 14 days. The results showed (Figure 5) that the effect of the RHC complex on hair growth was revealed after 7 days of administration. Compared with the blank group (0 mg/mL), hair growth was better in the 0.05–2 mg/mL RHC complex groups at 7 days, and at 14 days, the 0.05–2 mg/mL RHC complex had a better effect on hair growth and was close to the effect of the control group (5% minoxidil). Notably, the RHC complex at concentrations of 0.05 mg/mL, 0.1 mg/mL, and 2 mg/mL exhibited superior efficacy in promoting hair growth, suggesting a comparable effect to minoxidil.

### 3.6 The RHC complex promoted the expression of integrin, Laminin and Perlecan proteins

**FIGURE 6:**
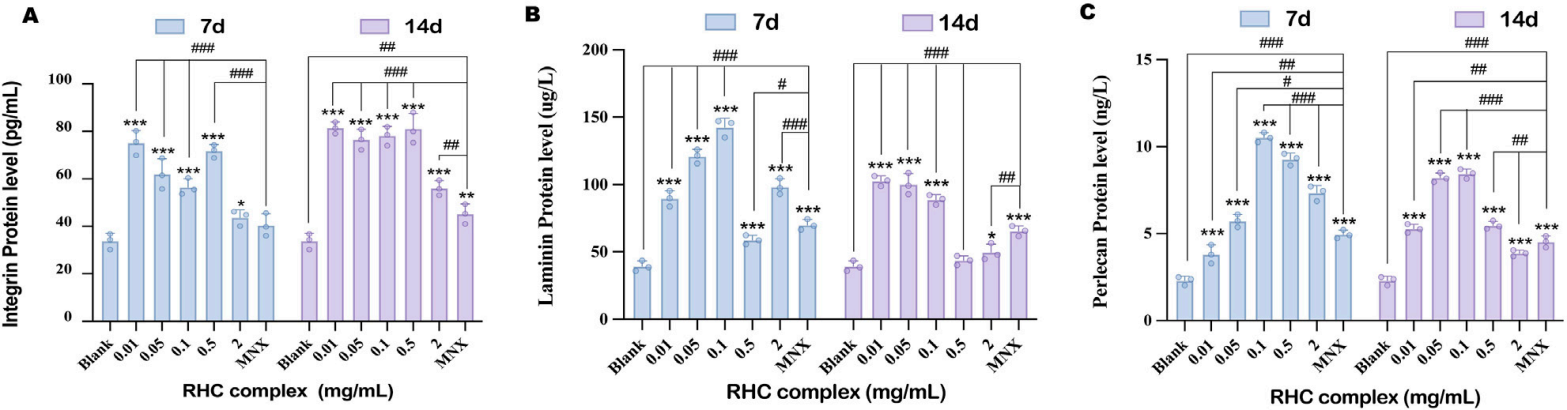
Expression of **(A)** Integrins, **(B)** Laminin, and **(C)** Perlecan proteins in the dorsal skin of rats in different concentrations of RHC complex-treated groups was examined by ELISA. RHC complex at 0 mg/mL was used as a blank group, and 5% minoxidil was used as a control group. The results are expressed as mean ± SD of three independent experiments. (*p < 0.05, **p < 0.01, ***p < 0.001 compared with the blank group; #p < 0.05, *##*p < 0.01, *###*p < 0.001 compared with the 5% minoxidil group).

**FIGURE 7:**
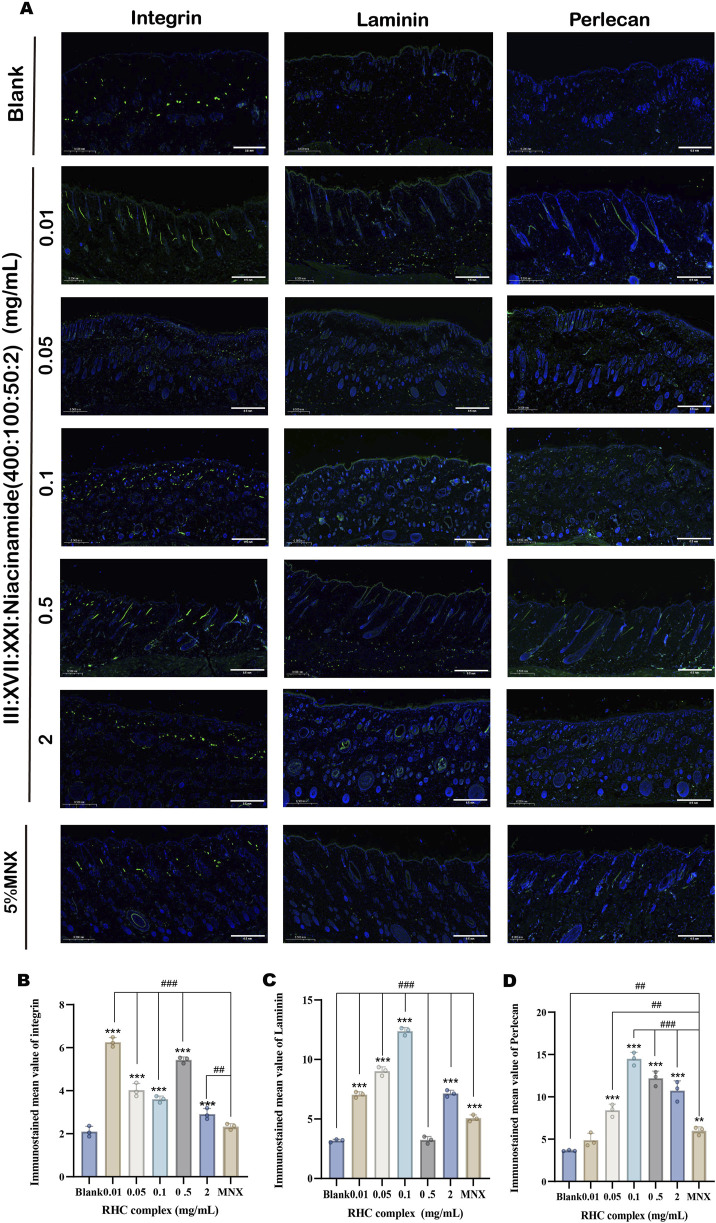
IF images of Integrin, Laminin and Perlecan protein expression in dorsal rat skin treated with different concentrations of RHC complex. **(A)** Images of isolated skin tissue IF labelled with Intergin (green), Laminin (green), Perlcean (green) and DAPI (blue). (Scale bar: 0.5 mm). **(B)** Quantitative analysis of Integrin. **(C)** Quantitative analysis of Laminin. **(D)** Quantitative analysis of Perlecan. The results are expressed as mean ± SD of three independent experiments. (*p < 0.05, **p < 0.01, ***p < 0.001 compared with the blank group; ##p < 0.01, ###p < 0.001 compared with the 5% minoxidil group).

Integrin, Laminin, and perlecan play crucial roles in hair growth and follicle development (Hayashi et al., 2002; Li et al., 2003; Gao et al., 2008; Kloepper et al., 2008; DeRouen et al., 2010; Park and Lee, 2021). The effects of the RHC complex on the expression of key proteins (Integrin, Laminin and Perlecan) in the extracellular matrix and cell surface were evaluated by ELISA (Gomes et al., 2002; Walma and Yamada, 2020; Jain et al., 2022; Jandl et al., 2022). The results showed (Figures 6A–C) that different concentrations of the RHC complex had promotional effects on the expression of the three proteins. When the treatment cycle was 7 days, the expression of Laminin and Perlecan increased with the concentration of RHC complex in the range of 0.01–0.1 mg/mL. The optimal expression levels were achieved at a concentration of 0.1 mg/mL, with Laminin reaching 142.55 ± 6.64 μg/L and Perlecan reaching 10.52 ± 0.28 ng/mL. However, when the concentration of RHC complex exceeded 0.1 mg/mL, the expression of these two proteins tended to decrease. In contrast, the expression of Integrin was highest within the concentration range of 0.01–0.5 mg/mL, with the optimal expression level of 75.24 ± 5.15 pg/mL observed at 0.01 mg/mL. When the dosing cycle was 14 days, the concentration of RHC complex in the range of 0.01–2 mg/mL promoted the expression of Integrin, Laminin and Perlecan. In addition, the overall expression promotion of the three proteins was stronger at a 7-day dosing cycle than at a 14-day dosing cycle, as shown by comparison. In order to further determine the effects of different concentrations of RHC complex on the expression of Integrin, Laminin and Perlecan in tissues, the dorsal skin of rats was collected for immunofluorescence staining after 7 days of the administration cycle and the results were analyzed quantitatively. The results showed (Figures 7A–D) that compared with the blank group (0 mg/mL) and 5% minoxidil, different concentrations of the RHC complex group showed the promotion effect on the three proteins, among which the concentration of 0.01–0.5 mg/mL of RHC complex promoted the expression of Integrin, Laminin and Perlecan after 7 days of dosing cycle. The best promotion effect on the expression of Integrin, Laminin and Perlecan was observed after 7 days of administration.

### 3.7 The RHC complex increased the number of hair follicles

**FIGURE 8:**
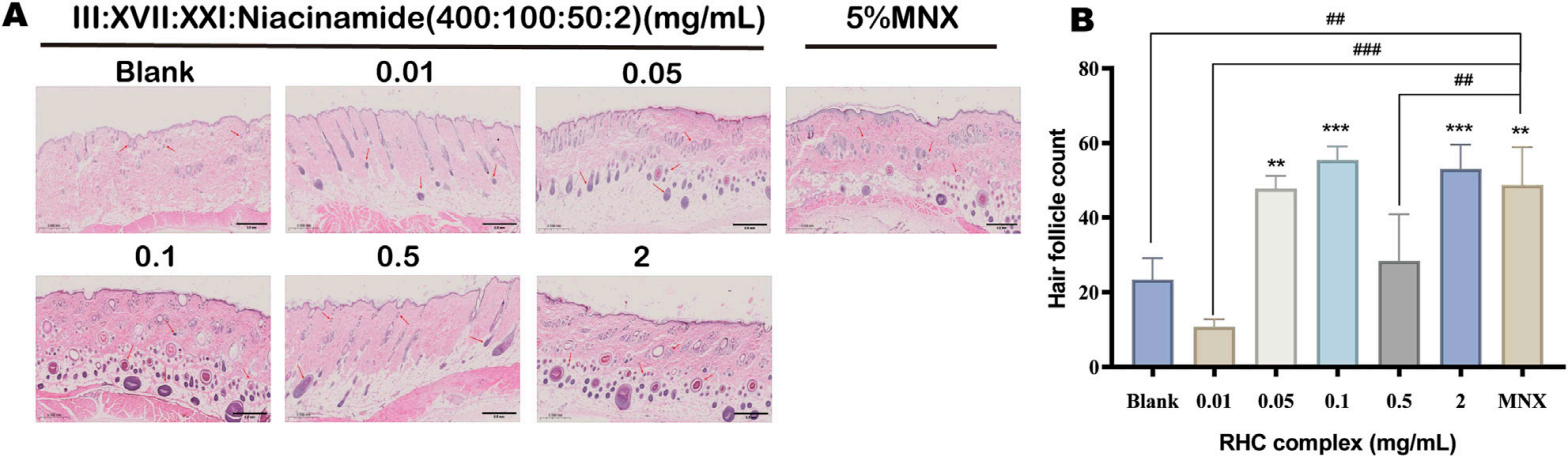
Effects of different concentrations of RHC complex (rhCOL III: rhCOL XVII: rhCOL XXI: niacinamide = 400:100:50:2) on hair growth (morphology and number) compared with 5% minoxidil and blank (0 mg/mL). **(A)** Representative images of longitudinal and transverse sections of H&E-stained skin tissue on day 7. (Scale bar: 0.5 mm). Red arrows in the images mark the hair follicles. **(B)** Number of hair follicles in each group of rats. The results are expressed as mean ± SD of three independent experiments. (**p < 0.01, ***p < 0.001 compared with the blank group; *##*p < 0.01, *###*p < 0.001 compared with the 5% minoxidil group).

Different concentrations of RHC complex (rhCOL III: rhCOL XVII: rhCOL XXI: Niacinamide = 400: 100: 50: 2) were applied to the hair removal sites of rats for 14 days, and after 7 days of treatment, the hair growth of rats was in a vigorous state. The changes in the morphology and number of hair follicles were detected and analyzed by HE histology, and the effects of the different concentrations of RHC complexes were compared with those of the control group and the blank group. Compared with the blank group, the hair follicles in the RHC complex treatment groups at different concentrations were in the anagen phase, the hair follicles became larger, longer, and densely arranged, the number of hair follicles increased, and the subcutaneous fat layer was thickened. The number of hair follicles in different concentrations of RHC complex was close to that of the control group when compared to the control group with 5% minoxidil (Figure 8A).

For a more visual comparison of changes in the number of hair follicles, the quantification of hair follicles in H&E sections was performed using statistics by photographing H&E stained skin cross sections of the samples at a magnification of ×100 (eyepiece ×10, objective ×10) and selecting the three areas with the highest number of hair follicles to be photographed. From the statistical results (Figure 8B), the number of hair follicles was higher in the RHC complex treated group at concentrations of 0.05 mg/mL, 0.1 mg/mL and 2 mg/mL and the subcutaneous adipose layer in the group was also thickened when compared with the blank and control groups. These results suggest that the RHC complex promotes hair growth to some extent and has the same effect as minoxidil (MNX), which is a representative drug approved by the U.S. Food and Drug Administration (US FDA) for the prevention of hair loss (Ohyama et al., 2010).

### 3.8 Physical quantitative evaluation for hair growth

**FIGURE 9:**
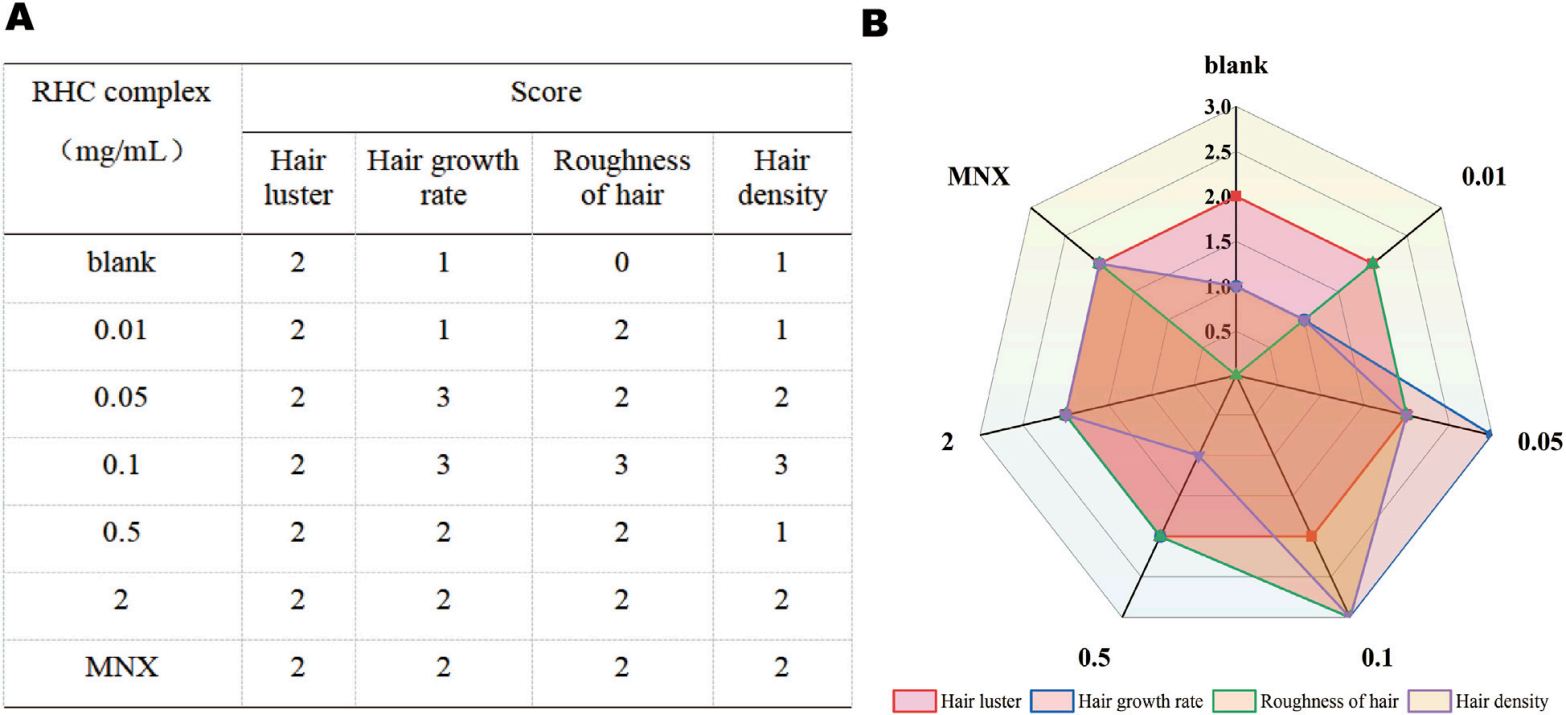
Physical quantification evaluation of hair in rats treated for 7 days, **(A)** Physical Ouantitative Assessment Growth. **(B)** Radar diagram for physical assessment ofhair growth.

To further describe the effect of RHC complex on hair state, we conducted a physical quantitative evaluation analysis (Figure 9). Different concentrations of RHC complex have no significant effect on hair luster, and the scores in each group are 2. In terms of hair growth rate, the RHC complex concentration within 0.05–2 mg/mL showed good hair growth rate compared to the blank group and scored higher than the 5% minoxidil and the rest of the concentration groups at concentrations of 0.05 mg/mL and 0.1 mg/mL, showing better growth rate. In terms of hair roughness, the 0.1 mg/mL group scored the highest and showed significant changes compared to the blank group, but the rest of the groups mostly had a score of 2 and did not show a continuous trend of 'getting smoother'. In addition, compared with other groups, the hair density of the RHC complex group with a concentration of 0.1 mg/mL also showed a higher score, reaching the highest value of 3, this indicates that the overall hair performance is better at a concentration of 0.1 mg/mL, and the effect of RHC complex on hair growth is not significantly concentration dependent.

## 4 Discussion

Currently, collagen is highly favoured in the cosmetics industry due to its unique biological properties. However, current applications face significant limitations, including safety risks associated with animal derived collagen and insufficient understanding of the synergistic mechanisms between collagen subtypes. Because of this, this study aims to explore the possibility of novel collagen applications, prepare a complex containing recombinant humanized collagen (RHC) of type XXI collagen, and study its effect on promoting hair growth using HFSCs model and WISTAR rat *in vitro* model. Our research results indicate that the RHC complex can improve hair growth and hair health.

Aging is an inevitable biological process that leads to noticeable manifestations in all skin tissues, including scalp skin and hair follicles (Liu et al., 2024). The hair follicle basement membrane (BM) rich in type IV collagen, laminin, and integrin provides the necessary mechanical support and signalling environment for the maintenance of HFSCs. As the core structural components of BM, laminin, type IV collagen, nestin, and heparin sulfate proteoglycans (HSPGs, including perlecan, aggrecans, etc.) jointly maintain tissue integrity (Jeong et al., 2020). Perlecan is a bridging protein polysaccharide between type IV collagen and laminin, which works synergistically with integrins (Lavorgna et al., 2023; Wang et al., 2025; Pozzi et al., 2017). Integrins are transmembrane heterodimers that mediate cell adhesion to bone marrow through specific ligand binding (laminin, fibronectin, collagen) (Pang et al., 2023). In this study, by combining ELISA and immunofluorescence analysis, we confirmed that the RHC complex upregulated the expression of adhesion proteins, integrins, and bead proteins in the BM layer of rat skin, structurally enhancing this microenvironment. This finding is consistent with the H&E staining observations.

HFSCs are the core of maintaining hair growth ability. They can secrete growth factors such as VEGF and FGF (Zografou et al., 2011), and promote angiogenesis and BM protein synthesis (such as Laminin and Perlecan), thereby maintaining the stability of hair follicle ecological niche. VEGF and trichohyalin are two indicators related to HFSCs, which are involved in regulating various signalling pathways of HFSCs. Zhang et al. confirmed that VEGF can protect HFSCs from androgen-induced apoptosis through the phosphatidylinositol 3-kinase (PI3K)/protein kinase B (AKT) pathway, and its protective effect is concentration dependent (Tobin, 2003). Transcription factor P63 plays a crucial role in skin development, mainly regulating cell self-renewal by modulating several components in signalling pathways, including Hedgehog (Hh), Notch, TGF - β/Smad, and WNT/β-catenin (Novelli et al., 2022). Overall, the levels of VEGF and p63 secreted by hair follicle stem cells after 8 and 16 h of cultivation with RHC complex were positively correlated with drug treatment concentration. This precisely indicates that the treatment of the RHC complex promotes the secretion of VEGF in HFSCs, successfully protecting HFSCs from androgen-induced-apoptosis. Trichohyalin is mainly expressed in the stratum corneum of hair follicles and is a protein related to hair structure and growth (Tobin, 2003). In 2003, Peter M. Steinert et al. pointed out that hair transparent protein enhances its mechanical strength in the inner root sheath by forming a stable cross-linked structure, which is crucial for hair growth and structure (Steinert et al., 2003). In addition, we also added β-integrin as a detection indicator (Ren et al., 2024). The research results showed that the secretion of hair transparent protein and β - integrin by cystic stem cells significantly increased after 8 and 16 h of cultivation with the RHC complex.

Compared with previous studies focusing on single collagen subtypes or isolated growth factors, the main innovation of this study is the formulation of a multi-component composite material that fully utilizes the unique but complementary biological effects of each component. Type XVII collagen, as a transmembrane collagen protein, stabilizes the HFSCs ecological niche through integrin-mediated adhesion and differentiation regulation (Matsumura et al., 2016). Τype III collagen enhances skin fibroblast proliferation and angiogenesis, synergistically enhancing hair follicle microenvironment vascularization. Type XXI collagen is a fiber-associated collagen protein with a discontinuous triple helix (FACIT) that enhances the integrity of the basement membrane (BM) through ECM remodeling (Kehlet and Karsdal, 2016). Another report suggests that the combination of type III collagen and type XVII collagen has shown a synergistic effect in promoting hair growth. In recent years, many scientists have been studying the effect of niacinamide on hair growth. Y. Choi et al. pointed out that niacinamide can protect hair follicle cells from oxidative stress damage by downregulating the expression of DKK-1, which helps maintain the health of hair follicles (Choi et al., 2021). On this basis, this study further expands its ideas by innovatively adding nicotinamide and type XXI collagen, exploring the possibility of synergistic effects on hair growth when RHC III, XVII, XXI and nicotinamide are mixed in a ratio of 400:100:50:2.

Importantly, the incorporation of nicotinamide, a precursor of NAD + with dual antioxidant and metabolic regulatory properties can enhance the proliferation ability of HFSCs (Kim et al., 2024), as evidenced by the dose-dependent increase in cell survival rate (Figure 1). Mechanistically, nicotinamide may alleviate the potential cytotoxicity of high-dose collagen exposure by protecting mitochondrial function and upregulating stress response pathways (such as Nrf2/ARE), while enhancing nutrient delivery by improving microcirculation (Olsen et al., 2002). Our systematic evaluation confirmed the biocompatibility and non-cytotoxicity of the composite material to HFSCs, laying the foundation for its application in the treatment of androgenic alopecia.

This study has pioneered a combination strategy centred around ECM, surpassing traditional single target hair growth methods. Our complex addresses the mechanical and biochemical aspects of follicular degeneration through synergistic coupling structure-ECM stabilization (via rhCOL III, rhCOL XVII, rhCOL XXI) and metabolic regulation (via nicotinamide). In addition, in order to improve the rigor of research, in the future, hair removal models should be optimized in research methods to reduce individual differences in animals and facilitate more accurate observation. Future research emphasis should be placed on optimizing the concentration and dosage form of the RHC complex, and exploring its long-term effects on hair growth and health. Prioritize exploring clinical synergies with existing therapies. These efforts will drive the translation of ECM targeted therapies into next-generation hair loss management solutions, bridging the gap between laboratory innovation and clinical impact.

## 5 Conclusion

In this study, the recombinant humanized collagen (RHC) complex composed of rhCOL III, rhCOL XVII, rhCOL XXI, and nicotinamide demonstrated significant potential in promoting hair growth and enhancing the stability of the hair follicle niche. Through *in vitro* experiments, the RHC complex exhibited non-cytotoxicity and improved the survival and functionality of hair follicle stem cells (HFSCs), with optimal effects observed at concentrations of 0.5–2 mg/mL after 16 h of treatment. The complex significantly upregulated key biomarkers, including VEGF, p63, trichohyalin, and β-integrin, which are critical for HFSCs proliferation, differentiation, and structural integrity of hair follicles. *In vivo* rat models further confirmed its efficacy, showing accelerated hair regrowth, increased hair follicle density, and enhanced expression of basement membrane (BM) proteins such as laminin, integrin, and perlecan, particularly at 0.05–2 mg/mL after a 7-day treatment cycle.

The RHC complex’s non-toxic profile, coupled with its capacity to augment HFSCs activity and function, renders it a potent candidate for hair growth research in clinical applications.

## Data Availability

The original contributions presented in the study are included in the article/Supplementary Material, further inquiries can be directed to the corresponding authors.

## Abbreviations

RHC, recombinant humanized collagen; MNX Minoxidil; RhCOL, III recombinant humanized type III collagen; RhCOL, XVII recombinant humanized type XVII collagen; RhCOL, XXI recombinant humanized type XXI collagen; ECM, extracellular matrix.
