# Supplementary material for: Recombinant humanized collagen combined with nicotinamide increases the expression level of rat basement membrane proteins and promotes hair growth
Source: Front Bioeng Biotechnol. 2025 Jun 12;13:1546779. doi: 10.3389/fbioe.2025.1546779 (PMC12198190; doi:10.3389/fbioe.2025.1546779)
Supplement: Supplementary file 1 [file Table1.docx]

| Number | Molecular weight | Amino Acid Sequence |
| --- | --- | --- |
| Col-3 | 10~43 kDa | GKSGDRGESG PAGPAGAPGP AGSRGAPGP QGPRGDKGE  TGERGAAGIK GHRGLE |
| Col-17 | 10~23.8 kDa | YVWSHPQFEK GSPGPKGDMG  SPPKGDRGF PGTPGIPGPL  GHPGPQGPKG QKGSVGDPGM  EGPGEKGERG AAGEPGPHGP  PGVPGSVGPK GSSGSPGPQG  PPGPVGLQGL RGEVGLPGVK  GDKGPMGPPG PKGDQGEKGP  PGPPGPPGPK GDQGPPGPRG  HQGEQGLPGF SGPPGPPGPQ  GPKGDKGDPG VPGALGIPGP  PGQKGEMGTP GPKGDRGPAG  PPGHPGPPGP RGHKGEKGDK  GDQHHHHH |
| Col-21 | 10~38 kDa | GKPGLQGPKGD PGLPGNPGYPG  QPGQDGKPGY QGIAGTPGVPG  SPGIQGARGLP GYKGEPGRDGD  KGDRGLPGFPG LHGMPGSKGE  MGAKGDKGSP GFYGKKGAKGE  KGNAGFPGLPG PAGEPGRHGKD  GLMGSPGFKGE AGSPGAPGQD  GTRGEPGIPGFP GNRGLMGQKG  EIGPPGQQGKK GAPGMPGLMG  SNGSPGQPGTP GSKGSKGEPGIQ  GMPGASGLKGE PGATG |
